# 

**Published:** 1877-03

**Authors:** 


### CONTENTS:

ARTICLE I.—The American Academy of Dental Surgery........................ 481
ARTICLE II.—The Physical History of Various Nations of the Earth with Special Reference to their Teeth. By John Allen, D. D. S. .......... 485
ARTICLE III—New York Odontological Society.................................. 495
ARTICLE IV.—Treatment of Teeth During Gestation and Lactation. By J. Williams, D. D. S......................................... 510
ARTICLE V.—Dental Education............................................. 515
ARTICLE VI.—Deaths from Chloroform....................................... 518

### EDITORIAL, ETC.

The Lost Arts.................................................... 519
Exercise for the Teeth........................................... 520
Acid Conditions of the Fluids of the Mouth....................... 521
Medical Degrees for Women........................................ 521

### MONTHLY SUMMARY.

Chemical Porcelain............................................... 522
A Fatal Case of Parotitis........................................ 523
Chloral Plaster in Neuralgia..................................... 524
Choice of Sedative for the Young or Aged......................... 524
Nervous Sensitiveness............................................ 525
Cold Water in Neuralgia.......................................... 526
Effects of Violent Pain on Animals and Men....................... 527
Poisonous Action of Salicylic Acid............................... 527
Excision of Nerves in Treatment of Spasmodic Contraction of Muscles.......... 528
Tincture of Aconite Root for Odontalgia.......................... 528

